# Two Different Transgenes to Study Gene Silencing and Re-Expression During Zebrafish Caudal Fin and Retinal Regeneration

**DOI:** 10.1100/tsw.2006.328

**Published:** 2006-12-15

**Authors:** Ryan Thummel, Christopher T. Burket, David R. Hyde

**Affiliations:** Center for Zebrafish Research and Department of Biological Sciences, University of Notre Dame, Notre Dame, IN, Indiana, USA

**Keywords:** zebrafish, retina regeneration, fin regeneration, transgenic, ef1-α, blastema, stem cells, histone H2A, DNA methylation

## Abstract

We used the 500-bp *Xenopusef1*-α promoter and the 2-kb zebrafish histone *2A.F/Z* promoter to generate several independent transgenic zebrafish lines expressing EGFP. While both promoters drive ubiquitous EGFP expression in early zebrafish development, they are systematically silenced in several adult tissues, including the retina and caudal fin. However, EGFP expression is temporarily renewed in the adult during either caudal fin or retinal regeneration. In the Tg(*H2A.F/Z:EGFP*) line, EGFP is moderately expressed in both the wound epithelium and blastema of the regenerating caudal fin. In the Tg(*ef1-α:EGFP*) line, EGFP expression is reinitiated and restricted to the blastema of the regenerating caudal fin and colabels with BrdU, PCNA, and *msxc*-positive cells. Thus, these two ubiquitous promoters drive EGFP transgene expression in different cell populations during caudal fin regeneration. We further analyzed the ability of the *ef1-α:EGFP* transgene to label nonterminally differentiated cells during adult tissue regeneration. First, we demonstrated that the transgene is highly methylated in adult zebrafish caudal fin tissue, but not during fin regeneration, implicating methylation as a potential means of transgene silencing in this line. Next, we determined that the *ef1-α:EGFP* transgene is also re-expressed during adult retinal regeneration. Specifically, the *ef1-α:EGFP* transgene colabels with PCNA in the Müglia, a specialized cell that is the source of neuronal progenitors during zebrafish retinal regeneration. Thus, we concluded that Tg(*ef1-α:EGFP*)nt line visually marks nonterminally differentiated cells in multiple adult regeneration environments and may prove to be a useful marker in tissue regeneration studies in zebrafish.

## INTRODUCTION

Among vertebrates, only certain species, such as specific urodele amphibians and teleost fishes, have the ability to regenerate multiple organs and tissue types. Recently, zebrafish caudal fin regeneration has emerged as an ideal model to further examine vertebrate regeneration due to the simpler anatomical structure of the caudal fin relative to the urodele limb[1]. While publications on teleost fin regeneration date back over 2 centuries[2,3,4], a functional and genetic understanding of fin regeneration remains fairly limited[5,6].

Forward genetic analysis has yielded only a small number of mutants with defects in fin regeneration[7,8,9,10,11]. Characterization of such mutants was limited to gross regeneration defects that could be visualized clearly because, until recently, transgenic lines that label specific cell types involved in fin regeneration did not exist; thus, mutants with subtle, but potentially important, defects were difficult to detect.

Reverse genetic techniques to study fin regeneration have also been limited by the techniques available for gene knock down in adult tissues and by the lack of *in vivo* markers to specific cell types. Use of certain pharmacological inhibitors, such as the *fgfr1* inhibitor, SU5402, have contributed to our knowledge as to what general signaling pathways are involved in fin regeneration[12]. From these studies, we know that Fgf signaling is required for blastema formation and that blastema markers, such as *msxc*, are downstream of this signaling cascade. However, the use of chemical inhibitors does have drawbacks. First, chemical inhibitors are not available for each gene of interest. Thus, our understanding of the hierarchy of candidate genes within a given signaling pathway, such as Fgfr1, is very limited. Second, without *in vivo* cell markers, a real-time analysis of fin regeneration is limited simply to growth reduction or fixing the tissue for histological or immunological analysis. This may lead to misinterpreting the identified role of a particular protein in fin regeneration.

We were, therefore, interested in generating transgenic zebrafish lines that express fluorescent markers in specific cell types during fin regeneration. We recently screened multiple transgenic lines that were designed to use a ubiquitous promoter (the *Xenopus ef1-$\alpha$* and *Danio rerio H2A/F.Z*) to express EGFP in the developing embryo but, for an unknown reason, were silenced in a variety of adult tissues, including the adult caudal fin. We anticipated that this type of line may re-express the transgene in the regenerating fin and provide a visual *in vivo* marker for the early responses during fin regeneration. This work describes two such lines, Tg(*ef1-$\alpha$:EGFP*)$^{nt}$ and Tg*(H2A.F/Z:EGFP)*$^{nt}$, that meet these criteria.

During fin regeneration, expression of the *H2A.F/Z:EGFP* transgene is expressed in both the wound epithelium and underlying mesenchyme, including the blastema. In contrast, expression of the *ef1-$\alpha$:EGFP* transgene is restricted to the mesenchyme, including the forming, proliferating, and distal-most blastema. EGFP expression in the Tg(*ef1-$\alpha$:EGFP*)$^{nt}$ line colabels with PCNA, BrdU, and *msxc* expression in the proliferative blastema. We also found the *ef1-$\alpha$:EGFP* transgene was re-expressed in the Müller glial cells during retinal regeneration, which serve as the source of neuronal progenitor cells during photoreceptor regeneration. Thus, the expression of the *ef1-$\alpha$:EGFP* transgene in both development and regeneration suggests that it may be a useful tool to visualize nonterminally differentiated cells and may serve as a powerful reagent to screen potential signaling molecules in the regeneration pathways of multiple tissues.

## RESULTS

### Generation of Tg(*ef1-$\alpha$:EGFP*)$^{nt}$ and Tg*(H2A.F/Z:EGFP)*$^{nt}$ Transgenic Fish Lines

The Tg(*ef1-$\alpha$:EGFP*)$^{nt}$ line was generated by coinjecting the pT2KXIG expression plasmid, which contains the 500-bp *Xenopus ef1-$\alpha$* promoter upstream of the EGFP reporter gene, with transposase mRNA into 1–4 cell stage zebrafish embryos. Similarly, the Tg*(H2A.F/Z:EGFP)*$^{nt}$ line was generated by cloning the 2-kb zebrafish *H2A.F/Z* promoter upstream of EGFP in pT2KXIG and coinjection with transposase mRNA into 1–4 cell stage zebrafish embryos. Six F1 carriers from both lines were identified by EGFP expression, raised and analyzed for EGFP expression at sexual maturity. EGFP-positive adults were out-crossed to the wild-type AB strain. Lines that produced ~50% of the offspring carrying the transgene were raised and analyzed for persistence of the transgene in subsequent generations. Four Tg(*ef1-$\alpha$:EGFP*)$^{nt}$ lines consistently produced 50% of offspring carrying the transgene and Southern analysis confirmed that two of these lines contained a single insertion (data not shown). Similarly, two

Tg*(H2A.F/Z:EGFP)*[nt] lines consistently produced 50% of the offspring carrying the transgene, suggesting that these contain single inserts. All of the single insertion lines have been maintained for six generations.

## The *ef1-α:EGFP* and *H2A.F/Z:EGFP* Transgene Expression Appears Ubiquitous, but is Silenced in Certain Tissues as Development Progresses

F2 embryos consistently demonstrated maternal expression of the *ef1-α:EGFP* transgene, as demonstrated by strong EGFP expression prior to the mid-blastula transition (Fig. 1A, n = 4 lines). One line exhibited a mosaic pattern of zygotic expression of the *ef1-α:EGFP* transgene in the F2 generation (data not shown), and was not analyzed further for this study. In the remaining three lines, strong, uniform zygotic expression of the transgene was observed in all cells by early somitogenesis (~10.5 hpf) (data not shown). This pattern and level of EGFP expression was maintained throughout the body and developing median fin fold until approximately 26 hpf (Fig. 1B). Over the next 96 h, EGFP expression of the transgene was observed in the body, but decreased in the developing fin fold (Fig. 1C).

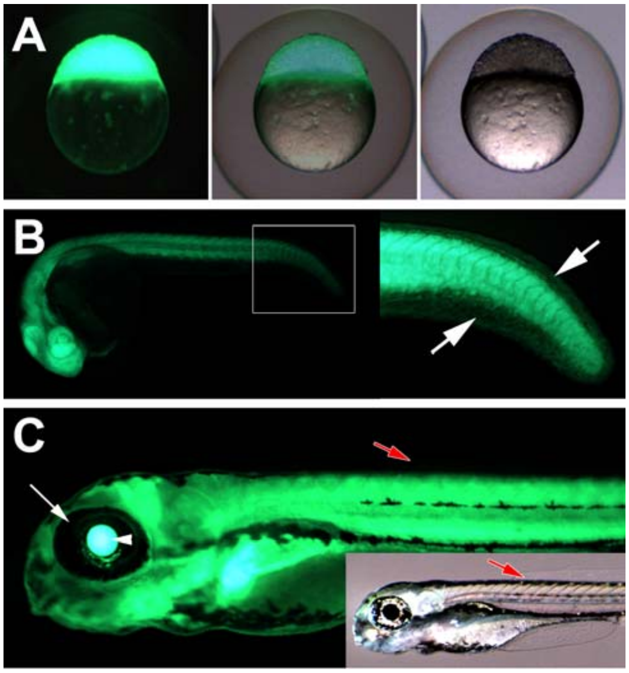

**FIGURE 1.** Expression of Tg(*ef1-α:EGFP)*[nt] during early development. (A) Maternal expression of the *ef1-α:EGFP* transgene through the blastula stage at 3 hpf. Fluorescent image on far left, brightfield image on far right, merge in the center. (B) Fluorescent image of a 26-hpf embryo (left panel) demonstrates broad expression of the *ef1-α:EGFP* transgene in the body. The boxed region is magnified to the right to show EGFP expression in the developing fin fold (arrows). (C) Fluorescent image of a 5-dpf embryo (with brightfield inset) demonstrates persistent EGFP expression in the body and lens (arrowhead), but reduced expression in the retina (white arrow) and fin fold (red arrow).

The expression of the *ef1-α:EGFP* transgene was similarly reduced in the developing retina (Fig. 2). Initially, strong expression was observed in the retinal neuroepithelium at 24 and 42 hpf (Figs. 2A and B). However, at 60 hpf, expression was dramatically reduced and nonuniform (Fig. 2C). And by 14 days postfertilization (dpf), *ef1-α:EGFP* transgene expression was below detectable levels in the retina (Fig. 2D), although it persisted in the lens.

Expression of the *H2A.F/Z:EGFP* transgene was generally less intense than the *ef1-α:EGFP* transgene (compare Figs. 1 and 3). F2 embryos again demonstrated maternal expression (data not shown, n = 2 lines). *H2A.F/Z:EGFP* expression was present in the body, retina, and lens through 48 hpf (Fig. 3A–D). EGFP expression was weakly detected in the developing fin fold at 26 hpf (Fig. 3A), but was absent by 48 hpf (Fig. 3B–D). At 4 dpf, low levels of *H2A.F/Z:EGFP* expression persisted in the retina (Fig. 3E). However, by 14 dpf, EGFP expression was only detected in the outer and inner plexiform layers (Fig. 3F). There was no significant EGFP expression in the adult retina (Fig. 3G).

To assess which tissues were less prone to silencing of the *ef1-α:EGFP* and *H2A.F/Z:EGFP* transgenes, we examined adult transgenic fish for the persistence or absence of EGFP expression (Table 1). We found that both transgenes were silenced in the adult retina (Figs. 2E and 3G), caudal fin (Fig. 4A and B), and heart atrium (Table 1). Additionally, the *ef1-α:EGFP* transgene, but not the *H2A.F/Z:EGFP* transgene, was silenced in the swim bladder and gills (Table 1). EGFP expression from both transgenes persisted in the body muscles, stomach, heart ventricle, and brain (Table 1). EGFP expression was generally stronger in the Tg(*ef1-α:EGFP*)$^{nt}$ line, especially in the body wall muscles (Table 1).

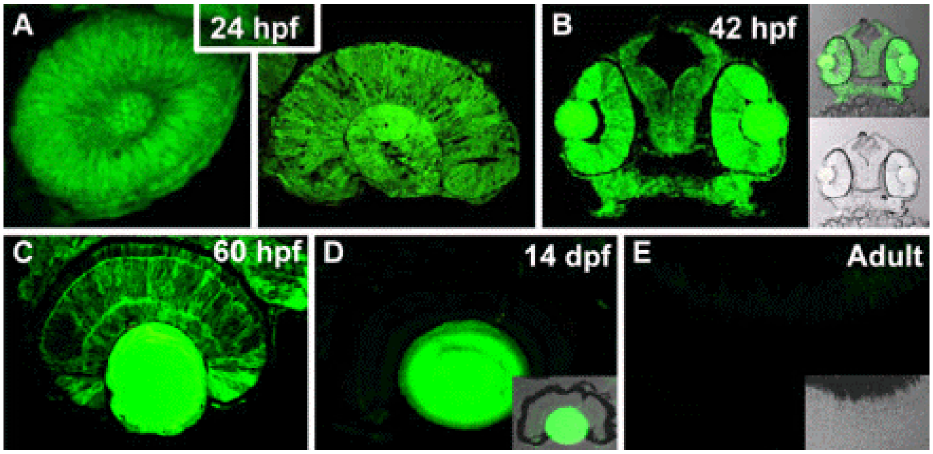

**FIGURE 2.** Expression of Tg(*ef1-α:EGFP*)$^{nt}$ during retinal development. Expression of the *ef1-α:EGFP* transgene is progressively silenced during retinal development. (A) Sagittal and frontal sections (left and right, respectively) taken at 24 hpf, shows *ef1-α:EGFP* expression in the developing lens and every neuroepithelial cell of the immature retina. (B) Frontal section shows ubiquitous expression of the *ef1-α:EGFP* transgene at 42 hpf. A brightfield image and a fluorescent/brightfield merged image are shown to the right. (C) Starting at 60 hpf, frontal sections differentially express the *ef1-α:EGFP* transgene in the retina, with continued strong expression in the lens. (D) By 4 dpf, expression of the *ef1-α:EGFP* transgene is primarily restricted to the lens and a few cells scattered throughout the retina. (E) By 14 dpf, *ef1-α:EGFP* transgene expression is completely silenced in the retina, but is still strong in the lens. A fluorescent/brightfield merge image is shown in the inset.

**TABLE 1**
**EGFP Expression in Adult Tissues**

| | CNS | Retina | Heart | Blood Vessels | Gills | Body Muscles | Stomach | Swim Bladder | Gonads | Fin |
|---|---|---|---|---|---|---|---|---|---|---|
| Tg(ef1α:EGFP) | + | – | +/– | – | – | ++ | ++ | – | + | – |
| Tg(H2A:EGFP) | + | – | +/– | – | + | + | ++ | + | + | – |

Key: –: no EGFP, +: EGFP, ++: strong EGFP: +/–: EGFP in the ventricle and bulbus, but no EGFP in the atrium.

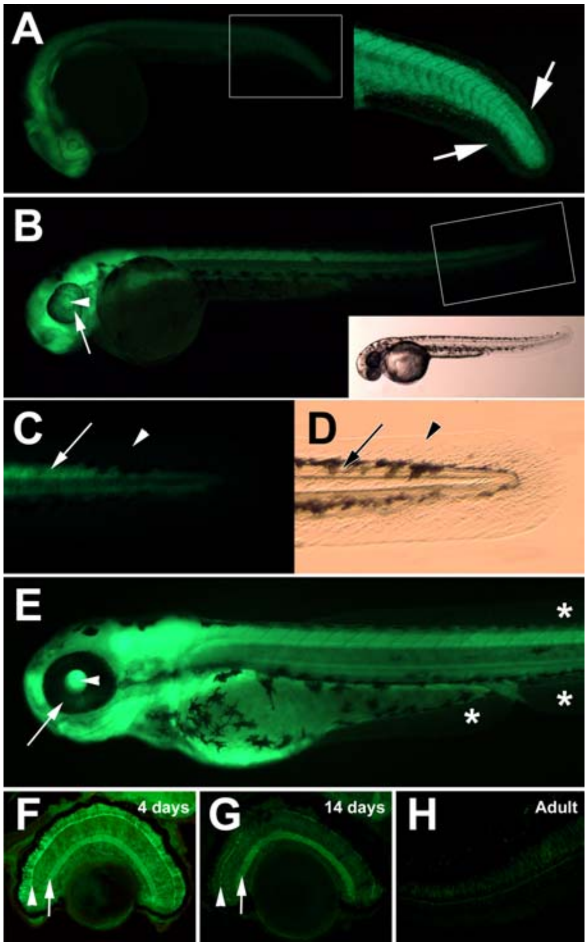

**FIGURE 3.** Expression of Tg(*H2A.F/Z:EGFP*)$^{nt}$ during zebrafish development. (A) A Tg(*H2A.F/Z:EGFP*)$^{nt}$ embryo showing uniform EGFP expression (left panel). The boxed region is magnified to the right and reveals the weak EGFP expression in the developing fin fold (right panel). (B) At 48 hpf, EGFP expression persists in the retinal neuroepithelium (arrow), and the developing lens (arrowhead). The boxed developing tail bud region is magnified in panels C and D. (C) Left panel, EGFP expression is observed in the developing tail bud (arrow), but not in the developing caudal fin fold (arrowhead). Right panel, brightfield image of left panel. (D) A transgenic embryo at 4 dpf shows persistent EGFP expression in the body and lens (arrowhead), reduced expression in the retina (arrow), and continued absence of expression in the fin folds (asterisks). (E) A frontal section from a 4-dpf embryo shows low-level ubiquitous expression of the *H2A.F/Z:EGFP* transgene in the retina. Elevated EGFP expression is present in the outer plexiform layer (OPL, arrowhead) and inner plexiform layer (INL, arrow). Expression in the lens is low, indicating that the lens expression observed in wholemount embryos (panel C, right) includes some refraction of the retinal expression. (F) A frontal retinal section at 14 dpf shows *H2A.F/Z:EGFP* transgene expression in the retina near background levels, except for the OPL and IPL (arrowhead and arrow, respectively). (G) Section from an adult Tg(*H2A.F/Z:EGFP*)$^{nt}$ shows an absence of transgene expression in the retina.

## Tg*(ef1-$\alpha$:EGFP)*$^{nt}$ and Tg*(H2A.F/Z:EGFP)*$^{nt}$ are Differentially Expressed During Caudal Fin Regeneration

Initial analysis of the Tg(*ef1-$\alpha$:EGFP*)$^{nt}$ and Tg*(H2A.F/Z:EGFP)*$^{nt}$ lines revealed that both do not exhibit significant EGFP expression in the adult fins, including the caudal fin (Fig. 4A and B). Higher-level microscopy revealed very weak EGFP expression in the bony fin rays near the fin girdle, which faded distally (Fig. 4A and B). This expression was so weak that it could only be observed at high magnification in those fin rays near the girdle that were not obscured by pigmentation.

By 24 h postamputation (hpa), both transgenes re-expressed EGFP in the regenerating fin (Fig. 4C and D). In the Tg*(ef1-a:EGFP)*$^{nt}$ line, EGFP expression was first visible in the presumptive-forming blastema and underlying mesenchymal compartment (Fig. 4E). Cryosections taken at 6 and 24 hpa confirm that the *ef1-α:EGFP* transgene was not expressed in the wound epithelium, but was restricted to the disorganized mesenchyme proximal to the amputation plane and to the overlying and forming blastema (Fig. 5B and C). During the first 96 hpa, there was a large increase in *ef1-α:EGFP* expression, during which time EGFP was clearly visualized in the blastema and trailing down to the site of the amputation (Fig. 4F). Following the turnover of the EGFP protein in the newly differentiated cells of the regenerate, by 7 dpa, *ef1-α:EGFP* expression was restricted solely to the blastema (Fig. 4I). A reduced level of EGFP expression persisted in this pattern through 30 dpa (Fig. 4J), even after fin regeneration appeared to be completed.

In the Tg*(H2A.F/Z:EGFP)*$^{nt}$ line, EGFP expression was visible by 24 hpa in a diffuse pattern throughout the regenerate (Fig. 4D and G). This diffuse expression was confirmed in cryosections taken at 6 and 24 hpa. The *H2A.F/Z:EGFP* transgene, unlike the *ef1-α:EGFP* transgene, was expressed in both the wound epithelium and forming blastema (Fig. 5E and F). The *H2A.F/Z:EGFP* expression increased throughout the first 96 hpa and was visualized throughout the newly-formed tissue (Fig. 4H). By 7 dpa, *H2A.F/Z:EGFP* expression was restricted to the proximal-most regenerate (Fig. 4K). Unlike the *ef1-α:EGFP* transgene, *H2A.F/Z:EGFP* expression slowly faded to near background levels by 14 dpa (data not shown) and was not detected at 30 dpa (Fig. 4L).

## The *ef1-α:EGFP* Transgene Coexpressed with PCNA, BrdU, and *msxc* at 48 hpa

We examined if the *ef1-α:EGFP* transgene was coexpressed with other known blastema and cell proliferation markers (PCNA, BrdU, and *msxc*) at 48 hpa in the regenerating caudal fin. The *ef1-α:EGFP* transgene was expressed in the distal-most and proliferative blastema (Fig. 6A and E). Similarly, PCNA immunolocalized to the proliferative region of the blastema (Fig. 6B). A merged confocal image of *ef1-α:EGFP* and PCNA immunolocalization revealed that the majority of the *ef1-α:EGFP*- and PCNA-positive cells colocalized (Fig. 6C and E). A medial compartment of cells located proximal to the most-proliferative blastema, however, contained cells that were solely PCNA positive, solely EGFP positive, and neither (Fig. 6D and insets). In addition, PCNA-positive cells in the epithelium were not EGFP positive (Fig. 6E, arrowhead), confirming that the *ef1-α:EGFP* transgene was not expressed in the epithelium. Finally, the distal-most blastema, which has been reported to contain a few cells that do not proliferate at this stage in regeneration[13], contained cells that expressed the *ef1-α:EGFP* transgene, but not PCNA (Fig. 6E, white arrows).

As an independent method to characterize the expression of the *ef1-α:EGFP* transgene relative to cell proliferation, BrdU incorporation experiments were performed in the Tg*(ef1-a:EGFP)*$^{nt}$ line. Caudal fins were partially amputated from adult Tg*(ef1-a:EGFP)*$^{nt}$ zebrafish and allowed to regenerate for 47.5 hpa, at which point BrdU was injected intraperitoneally. To label only the newly-most formed cells, fins were harvested 30 min later, and wholemount immunolocalization of BrdU was determined. Similar to the results obtained with the PCNA localization, we found many BrdU-positive cells in the proliferative blastema that also expressed the *ef1-α:EGFP* transgene (Fig. 6F).

To independently demonstrate that the *ef1-α:EGFP* transgene colabeled with blastema markers, wholemount *in situ* hybridization for *msxc* expression was performed on the Tg*(ef1-a:EGFP)*$^{nt}$ line at 48 hpa (Fig. 6G). Although this procedure squelched EGFP visualization, immunocytochemistry using a monoclonal EGFP antibody demonstrated that the *msxc* transcript colabeled with the *ef1-α:EGFP* transgene expression (Fig. 6H).

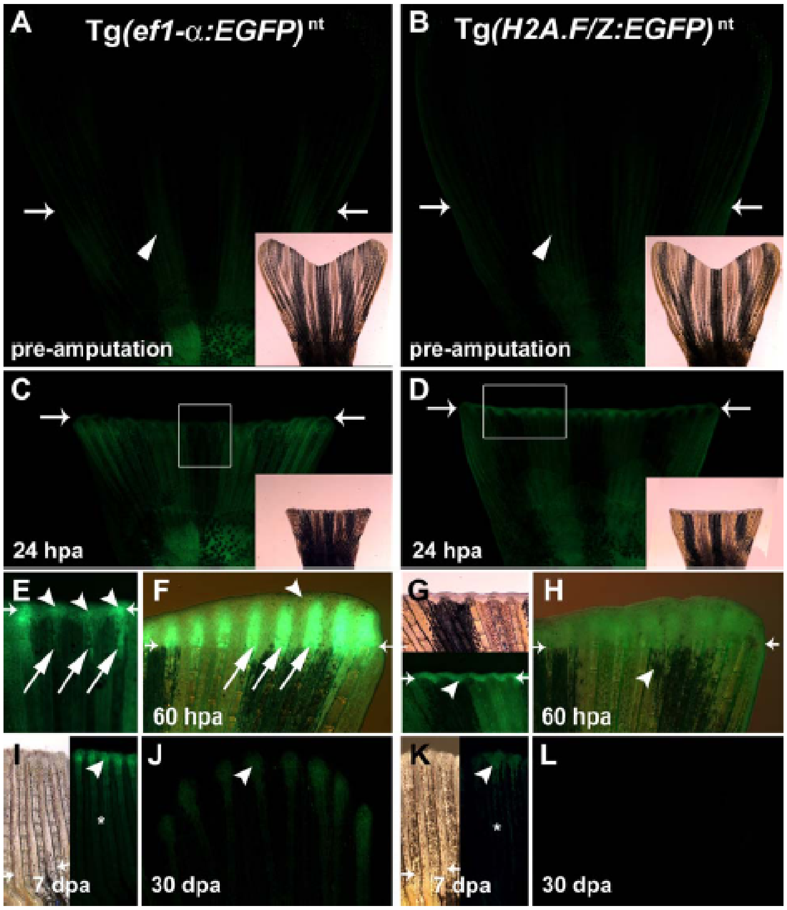

**FIGURE 4.** Differential expression of Tg*(ef1-a:EGFP)*nt and Tg*(H2A.F/Z:EGFP)*nt during caudal fin regeneration. Panels on the left (A, C, E, F, I, J) represent Tg*(ef1-a:EGFP)*nt fins, while panels on the right (B, D, G, H, K, L) represent Tg*(H2A.F/Z:EGFP)*nt fins. Wholemount fluorescent images reveal very low levels of EGFP expression in both the Tg*(ef1-a:EGFP)*nt and Tg*(H2A.F/Z:EGFP)*nt fins (panels A and B, respectively). A single white arrowhead denotes very weak EGFP expression, which can only be observed at high magnification in nonpigmented areas of the proximal fin, which is not observed distally. Two white arrows flanking the fin denote the plane of amputation used for the regeneration studies shown in the following panels. A brightfield image of both fins is shown in the inset. (C,D) Fluorescent images of the transgenic fins shown in panels A and B at 24 hpa (with brightfield images inset). Both transgenic fins reveal an up-regulation of EGFP expression. (E) Close-up of the boxed region outlined in panel C, revealing expression of the *ef1-a:EGFP* transgene in the presumptive blastemas (arrowheads) and in a few cells (large arrows) proximal to the amputation plane (small arrows). (F) A merged fluorescence/brightfield image at 60 hpa shows intense EGFP expression in the blastemas and trailing to the amputation plane (small arrows). Note the absence of EGFP expression in the wound epithelium (arrowhead). (G) Brightfield and fluorescent images of the boxed region in panel D, reveals EGFP expression from the *H2A.F/Z:EGFP* transgene at 24 hpa. Note the diffuse EGFP expression throughout the newly formed tissue (arrowhead). (H) A merged fluorescence/brightfield image at 60 hpa shows diffuse EGFP expression from the *H2A.F/Z:EGFP* transgene throughout the newly formed tissue. (I) Brightfield and fluorescence images at 7 dpa demonstrate that EGFP expression is restricted to the distal-most mesenchyme (arrowhead), but is no longer present in the differentiated regenerated fin tissue (asterisk). (J) Fluorescent image at 30 dpa shows persistent low-level expression at the distal-most fin. (K) Brightfield and fluorescence images at 7 dpa demonstrate that EGFP expression is now restricted to the tissue at the distal-most aspect of the fin (arrowhead), but is no longer present in the differentiated regenerated fin tissue (asterisk). (L) Fluorescent image at 30 dpa shows the absence of expression.

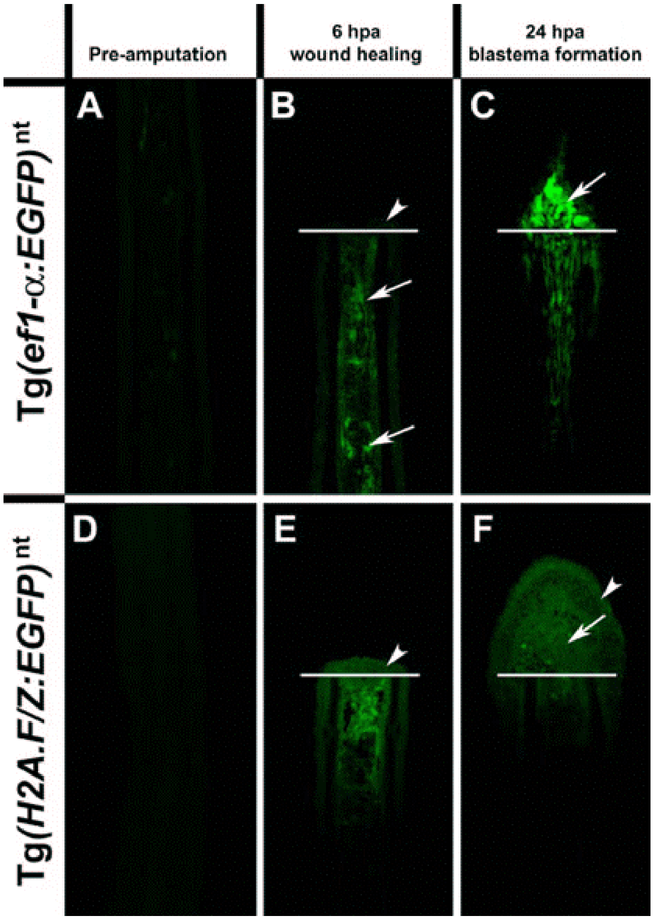

**FIGURE 5.** Tg*(ef1-a:EGFP)*^nt and Tg*(H2A.F/Z:EGFP)*^nt are expressed in different cell populations during caudal fin regeneration. Sections from Tg*(ef1-a:EGFP)*^nt and Tg*(H2A.F/Z:EGFP)*^nt fins demonstrate the level and location of EGFP expression. (A) Prior to fin amputation, EGFP was not detected in the distal fin. (B) During wound epithelium formation (6 hpa), EGFP is weakly expressed in mesenchymal cells (arrows) proximal to the amputation plane (represented by the white line), but is not detected in the wound epithelium (arrowhead). (C) During blastema formation (24 hpa), EGFP expression is intense in the developing blastema (arrow) and weakly expressed in the disorganized mesenchyme proximal to the amputation plane (represented by the white line). EGFP is not detected in the surrounding wound epithelium. (D) Similar to Tg*(ef1-a:EGFP)*^nt, EGFP is not detected in the distal region of Tg*(H2A.F/Z:EGFP)*^nt fins prior to amputation. (E) During wound healing, EGFP is weakly expressed in the wound epithelium (arrowhead) and underlying mesenchyme. (F) During blastema formation, EGFP is diffusely expressed at moderate levels in both the forming blastema (arrow) and surrounding wound epithelium (arrowhead).

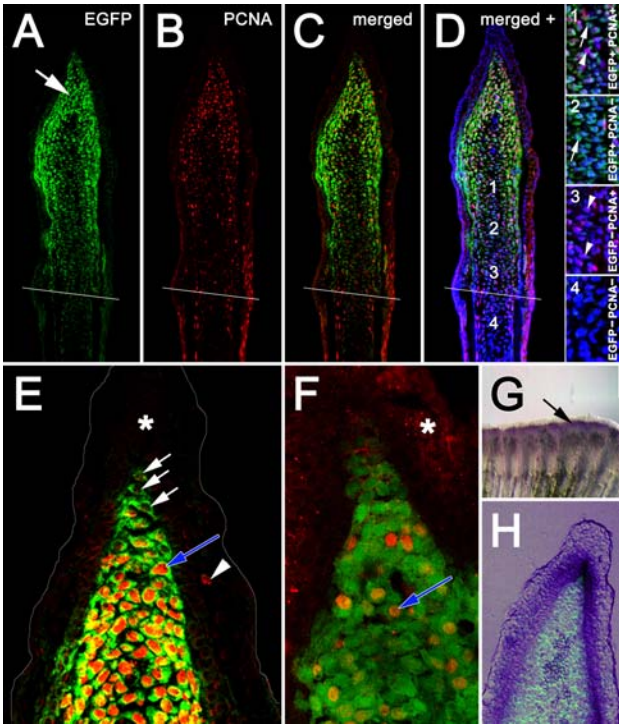

**FIGURE 6.** The *ef1-a:EGFP* transgene colabels with PCNA, BrdU, and *msxc* at 48 hpa. Confocal fluorescent images of a single cryosection from a Tg*(ef1-a:EGFP)*$^{nt}$ fin at 48 hpa demonstrate coimmunolocalization of EGFP and PCNA. (A) Immunolocalization of EGFP in the regenerating Tg*(ef1-a:EGFP)*$^{nt}$ fin is restricted to the mesenchyme, including the blastema (arrow). The location of the amputation plane is marked with a line. (B) PCNA immunolocalization (red) on the same section shown in panel A is also detected in the mesenchyme. (C) A merged image of panels A and B, reveals the large overlap of EGFP- and PCNA-positive cells. (D) The merged fluorescent image of panel C is colabeled with TO-PRO-3 (shown in blue), a nuclear stain. Numbers correspond to different regions containing different populations of cells that are shown in the insets on right. Panel 1 shows a mixed population of EGFP- (arrows) and PCNA-positive (arrowheads) cells. Panel 2 reveals a population of cells that are primarily only TO-PRO-3 and EGFP positive (arrows). Panel 3 shows a group of cells that are primarily TO-PRO-3 and PCNA positive (arrowheads). Panel 4 outlines a group of cells that are primarily TO-PRO-3 positive, but neither EGFP nor PCNA positive. (E) Close-up of a merged confocal image reveals EGFP and PCNA coimmunolabeling. The asterisk denotes the wound epithelium, which does not contain EGFP-positive cells. The distal-most blastema contains a few cells that are EGFP positive, but PCNA negative (white arrows). The proliferative blastema, just proximal to these cells, contains many cells that are EGFP and PCNA positive (blue arrow). A PCNA-positive cell in the wound epithelium (white arrowhead) is EGFP negative. (F) Confocal image of wholemount immunolabeling for BrdU (red) and EGFP expression from the Tg*(ef1-a:EGFP)*$^{nt}$ regenerating fin. The asterisk denotes the wound epithelium, which is EGFP negative. The blue arrow indicates one of the many BrdU- and EGFP-positive cells. (G) Wholemount *in situ* hybridization *msxc* expression in the regenerating Tg*(ef1-a:EGFP)*$^{nt}$ caudal fin. (H) Cryosection of the regenerating caudal fin shown in panel G demonstrating *msxc* expression relative to EGFP immunolocalization. Note that many EGFP-positive cells (green) colabel with *msxc* (purple).

## The Regenerating Caudal Fin Contains both Methylated and Unmethylated States of the *ef1-$\alpha$:EGFP* Transgene

We tested whether *de novo* DNA methylation was a potential mechanism of the silencing and re-expression of the *ef1-$\alpha$:EGFP* transgene in fin regeneration. It was previously shown that EGFP is highly methylated in silenced transgenic mice, and that this methylation can spread to surrounding promoters and transgenes[14,15,16]. In the regenerating zebrafish fin, the *ef1-$\alpha$:EGFP* transgene is silenced in the epithelium and only expressed in the underlying blastema tissue. We hypothesized that if methylation was playing a role in the differential expression of the transgene in these two tissues, then the regenerative tissue would contain the transgene in both methylated (silenced) and nonmethylated (not silenced) states. At 4 dpa, the entire regenerative fin tissue (epithelium and blastemas) was harvested and tested for DNA methylation using the standard technique of bisulfite sequencing. Specifically, we analyzed 19 CpG sites surrounding the start codon of the EGFP. We found that the regenerating fin tissue contained two different methylation patterns (Fig. 7), highly methylated (15 or more of the 19 CpG sites methylated) and highly nonmethylated (4 or fewer of the 19 CpG sites methylated). In an independent PCR amplification with different PCR primers, we identified additional highly methylated clones. The methylated CpG sites in the highly nonmethylated clones varied (Fig. 7), making it difficult to determine if any specific sites are potentially critical for transgene expression or silencing. However, the overall level of methylation appeared dramatic.

## The *ef1-α:EGFP* Transgene is Expressed in Dividing Müller Glia, the Source of Neuronal Precursors in the Regenerating Zebrafish Retina

We tested whether the *ef1-α:EGFP* transgene was re-expressed in other regenerative tissues besides the caudal fin. Zebrafish have the ability to regenerate their retinas following a variety of insults, such as visible light[17,18], heat[19], neurotoxins[20,21,22,23,24], or surgical lesion[25,26]. Although each model results in the loss of a specific subset of neuronal classes, a specialized glial cell, called the Müller glia, serves as a source of neuronal progenitors for regeneration of lost retinal neurons[27,28,29].

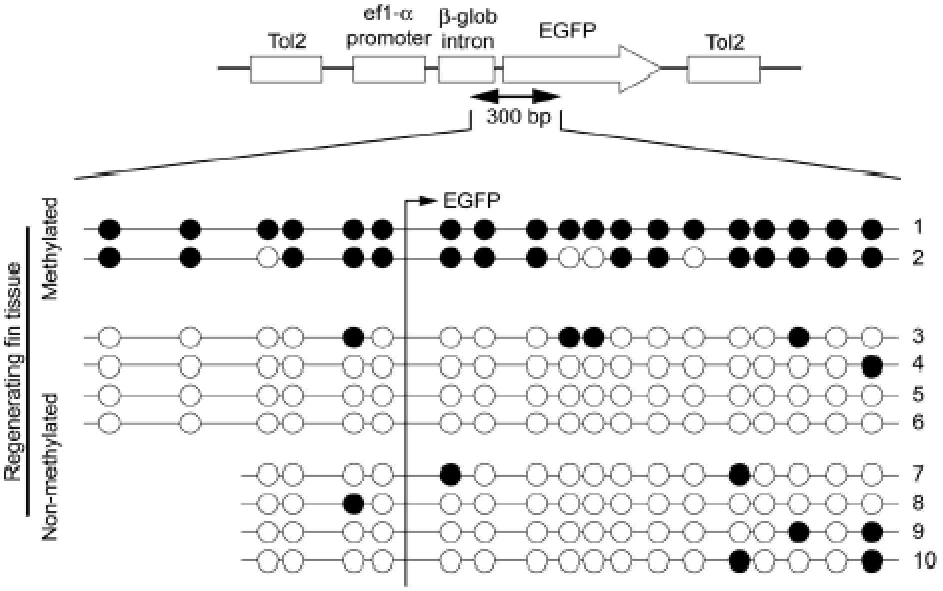

**FIGURE 7.** The regenerating caudal fin contains both methylated and unmethylated states of the *ef1-a:EGFP* transgene. Methylation pattern of a region of the *ef1-a:EGFP* transgene during fin regeneration. A schematic of the *ef1-a:EGFP* transgene in the Tol2 pT2KXIG element is shown above, with the region of the EGFP reporter gene that was analyzed shown below. DNA samples were obtained from the regenerative tissue of a 4-dpa fin. Two different primer sets were used to amplify an approximately 300-bp region surrounding the EGFP translational start site (marked by an arrow). Each line represents a different PCR subclone; subclones 1–6 were obtained using primer set 1 and clones 7–10 using primer set 2. The state of methylation of each subclone was analyzed using bisulfite conversion of nonmethylated cytosines to uracils and DNA sequencing. Each of the 19 CpG methylation sites in the first primer set region and 17 CpG sites in the second primer set region is represented by a small circle. Notice that both primer sets examine 17 common CpG sites. Empty circles indicate an absence of methylation, while closed circles indicate that the CpG sequence was methylated. Note that two different states of methylation were observed, a high level of methylation in subclones 1 and 2 (top) and a low level of methylation in subclones 3–10 (bottom).

We used the light-lesion model to study the loss and regeneration of photoreceptors in the *albino* Tg*(ef1-a:EGFP)*$^{nt}$ line. We previously demonstrated that the Müller glia re-enter the cell cycle within 31 h of light treatment to generate inner nuclear layer (INL) neuronal progenitor[29], which ultimately give

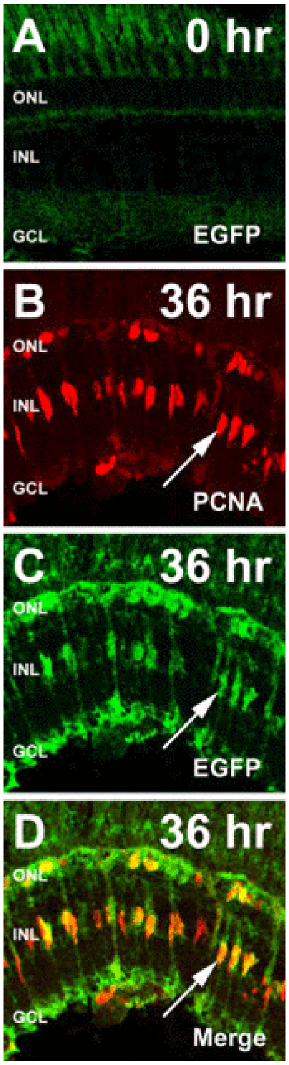

**FIGURE 8.** The *ef1-a:EGFP* transgene is expressed in dividing Müller glia, the source of neuronal progenitor cells in the regenerating zebrafish retina. Dark-adapted adult *albino* Tg*(ef1-a:EGFP)*$^{nt}$ were subjected to light treatment for either 0 h (panel A) or 36 h (panels B–D). Retinas were subsequently harvested and processed for immunolocalization of EGFP and PCNA. (A) At 0 h of light treatment, no EGFP expression from the *ef1-a:EGFP* transgene was observed in the retina. (B–D) Following 36 h of light treatment, PCNA-positive INL layer cells are observed (panel B), which colabel with the EGFP expressed from the *ef1-a:EGFP* transgene (panels C and D). The EGFP-expressing cells were determined to be Müller glia based on their morphology, which contain processes that extend from the ganglion cell layer to the outer limiting membrane (panel C), and that the Müller glia are the only PCNA-labeled cells in the INL at this time in regeneration[29]. INL, inner nuclear layer; ONL, outer nuclear layer; GCL, ganglion cell layer.

rise to both rod and cone photoreceptors[17]. As shown in Fig. 8, the *ef1-α:EGFP* transgene was not expressed in the adult retina (Fig. 8A). However, following 36 h of light treatment, EGFP was re-expressed in the Müller glial cells (Fig. 8C). The identity of the EGFP-positive cells as Müller glia was based on their location in the INL, their processes that extend to both the ONL and GCL, and their coexpression of GFAP (data not shown). Further, the EGFP-positive Müller glial cells colabeled with PCNA (Fig. 8D), which is consistent with the *ef1-α:EGFP* transgene expression in the dedifferentiated neuronal progenitor cells in the regenerating adult retina.

## DISCUSSION

Zebrafish is a powerful vertebrate model for the genetic analysis of a variety of developmental processes. In addition, zebrafish have recently become one of the preferred models for the study of tissue regeneration. In particular, there are multiple studies in zebrafish that have focused on regeneration of retinas[17,30], heart tissue[13], and fins[11,31,32,33]. Although researchers utilizing zebrafish have a wider range of genetic tools to study regeneration than many other regeneration model systems, such as the planarian, salamander, and newt, many of the genetic tools routinely used in flies and mice are either currently unavailable or just being developed for use in zebrafish. For example, genetic tools that could be applied to zebrafish regeneration include *in vivo* electroporation of morpholinos into the regenerating zebrafish fin to knock down the expression of a specific protein[34], the application of Cre-lox for site-specific recombination[35], and siRNA[36]. The generation and use of cell-specific markers and transgenic lines expressing cell-specific reporters to study regeneration is another area that is needed in zebrafish.

This work describes two lines, Tg(*ef1-α:EGFP*)$^{nt}$ and Tg*(H2A.F/Z:EGFP)*$^{nt}$, that up-regulate EGFP expression in specific cell populations during fin regeneration that could be used — independently or in conjunction with other available genetic tools — to mark particular cell types during the various stages of zebrafish caudal fin regeneration. Following partial amputation of the caudal fin, the first stage in zebrafish fin regeneration is wound epithelium formation. At a regeneration temperature of 33$^{o}$C, a thin layer of epithelium forms over the wound by approximately 6 hpa. At this stage in regeneration, expression of the *H2A.F/Z:EGFP* transgene is expressed in both the wound epithelium and underlying mesenchyme, but not in mature epithelium or mesenchyme (Fig. 5E). During wound epithelium formation, an unknown signal or signals trigger the disorganization of the underlying mesenchyme[37,38], which is followed by osteoblasts and fibroblasts re-entering the cell cycle[8]. Interestingly, during this stage of regeneration, the expression of the *ef1-α:EGFP* transgene is up-regulated in a few mesenchyme cells proximal to the amputation plane (Figs. 4 and 5), which likely represent the osteoblasts and fibroblasts that are re-entering the cell cycle. The dedifferentiated osteoblasts and fibroblasts rapidly divide, and by 24 hpa, a proliferative blastema has formed distal to each bony fin ray. During this stage of regeneration, the *H2A.F/Z:EGFP* transgene continues to be diffusely expressed in both the wound epithelium and underlying blastema (Figs. 4H and 5F). Expression of the *ef1-α:EGFP* transgene, in contrast, is restricted to the proliferating blastema and a few mesenchyme cells proximal to amputation plane (Fig. 5C). During fin outgrowth, beginning by 48 hpa, the blastema is subdivided into a distal-most, nonproliferative blastema, composed of only a few cells, and a proximal, proliferative blastema. During this stage, the *ef1-α:EGFP* transgene is expressed in both the distal-most and proliferating blastema. This was confirmed by *ef1-α:EGFP* coexpression with PCNA and *msxc* in the proliferative blastema (Fig. 6).

Taken together, these data suggest that the *H2A.F/Z:EGFP* transgene marks newly formed wound epithelium and mesenchyme during fin regeneration, while the expression of *ef1-α:EGFP* transgene may be a useful tool to visualize nonterminally differentiated cells in regenerative tissues. In support of this, we found *ef1-α:EGFP* expression in the distal-most mesenchyme in SU5402 fins, even though fin outgrowth was completely inhibited (data not shown). In addition, we demonstrated that the *ef1-α:EGFP* transgene was also re-expressed in the dividing Müller glia cells of the regenerating retina (Fig. 8), which serve as the source of neuronal progenitors for regeneration of lost retinal neurons[27,28,29]. It is important to note that fin regeneration utilizes a blastema-based model of regeneration and regeneration of the light-damaged retina uses Müller glial cells as a source of progenitors. However, in both cases, the *ef1-α:EGFP* transgene colabeled with PCNA-positive progenitor cells.The mechanism of action behind transgene silencing in these lines is currently unclear. One potential mechanism is *de novo* DNA methylation of CpG sequences in the transgene. Recently, an elegant study in the mouse model compared the DNA methylation of a *ROSA26:EGFP* transgene to the endogenous *ROSA26* DNA located on chromosome 6[16]. High levels of methylation were found in the transgene, but low levels were detected in the endogenous promoter region[16]. Because both the EGFP coding sequence and the adjacent *ROSA26* promoter were highly methylated, the authors suggested that the EGFP acted as a methylation target that spread to the adjacent *ROSA26* promoter[16]. A different group recently reported that the presence of a *PGK:EGFP* transgene silenced an adjacent *LCR:*β*-globin* transgene[14]. Interestingly, when these authors used a modified EGFP that lacked CpG methylation sites, *LCR:β-globin* expression was partially restored[14]. Similarly, we analyzed regenerating zebrafish fin tissue, which contains wound epithelium, where the transgene is silenced, and regenerative blastemas, where the transgene is expressed. We found both methylated and unmethylated forms of the *ef1-α:EGFP* transgene in the regenerating fin (Fig. 7). Based on these data and the previous data from the mouse, we hypothesize that DNA methylation and demethylation plays a roll in transgene silencing and re-expression during tissue regeneration.

Although there are many potential uses for these lines, we intend to use them in a subtractive gene microarray experiment designed to reveal cell- and temporal-specific expression of transcripts during fin and retinal regeneration. A previous work describes subtractive hybridization and differential display screens for genes expressed at two time points in zebrafish caudal fin regeneration[6]. Unfortunately, the inability to separate the wound epithelium from the blastema forced the authors to perform *in situ* hybridizations on a subset of the genes to determine their expression pattern[6]. The transgenic lines described in this work could be used to isolate different cell populations with a fluorescent-activated cell sorter. The corresponding RNAs could then be used in a gene microarray experiment from only a specific subset of cells during the various stages of regeneration. For example, the EGFP-positive cells from the Tg(*H2A.F/Z:EGFP*)$^{nt}$ line during the latter stages of fin outgrowth would express genes involved in the continuation of fin outgrowth, but not those genes involved in the differentiation of newly formed tissue. Conversely, isolation of EGFP-positive cells from the Tg(*ef1-α:EGFP*)$^{nt}$ line would express genes involved in blastema formation (at 6–24 hpa) and maintenance (at 48 hpa), while eliminating genes involved in wound epithelium formation and tissue differentiation. Thus, it is feasible that one could design a subtractive microarray experiment from which the gene sets obtained from the entire fin, entire regenerate, *H2A.F/Z:EGFP*- and *ef1-α:EGFP*-positive cells (at various stages of regeneration), would reveal a matrix of genes essential to each stage of caudal fin regeneration. Further, by comparing these data to the microarray data sets from regenerating zebrafish retinas[27,29], genes that are essential for general tissue regeneration could be sorted from genes that are essential to regeneration of a specific organ.

## MATERIAL AND METHODS

### Generation of Transgenic Lines

To generate transgenic fish containing the EGFP gene under the *Xenopus ef1-α* minimal promoter, we used the pT2KXIG plasmid that contains the 500-bp *ef1-α* promoter upstream of EGFP within a nonautonomous Tol2 transposable element[39]. The plasmid was purified using the Qiagen Maxi Prep Kit (Qaigen; Valencia, CA), followed by phenol/chloroform (1:1) extraction. Purified plasmid (25 ng/μl) was coinjected with *in vitro* transcribed Tol2 *transposase* mRNA (25ng/μl) into 1–4 cell stage embryos. The mRNA was produced as previously reported[35]. Embryos expressing EGFP were isolated and raised to adulthood, at which point they were out-crossed to AB (wild-type) adults to determine if any were founder fish that contained the *ef1-α:EGFP* transgene in their germline. Founder fish were then mated several times to generate families of F1 fish that were used in this study.

To generate transgenic fish containing the EGFP gene under the histone variant promoter (*H2A.F/Z*), we acquired the *H2A.F/Z:EGFP* transgene[40]. To clone the *H2A.F/Z* promoter into the Tol2 EGFP backbone, the following changes were made to the *H2A.F/Z-EGFP* plasmid. First, the *Kpn*I site in the *H2A.F/Z-EGFP* plasmid was changed to a *Xho*I by *in vitro* mutagenesis using the oligo 5’-CTCGAGGTAC-3’. Second, the *Nhe*I site was changed to a *Sal*I restriction site by *in vitro* mutagenesis using the oligo 5’-CTAGGTCGAC-3’. Using these newly introduced restriction sites, the *H2A.F/Z* promoter was subcloned into the *Xho*I-*Sal*I–digested pT2KXIG vector and replaced the *ef1-α* promoter. It should be noted that this promoter contains a portion of the 5’ UTR of the *H2A.F/Z* gene, which did not prevent EGFP expression. This plasmid was then purified and coinjected with the *in vitro* transcribed

Tol2 *transposase* mRNA as described above. Founder fish containing the *H2A.F/Z:EGFP* transgene were isolated and mated as described above.

## Fish Husbandry and Collection

All transgenic zebrafish were maintained in the Center of Zebrafish Research at the University of Notre Dame under a 14:10 h light:dark cycle at 28.5°C[41,42]. Fish were fed three times daily a combination of dry food and brine shrimp. Embryos were collected immediately following fertilization (0–0.5 hpf) and maintained at 28.5°C. To increase the rate of regeneration approximately twofold, the fish were maintained at 33°C after caudal fin amputation. Earlier studies demonstrated that the regeneration mechanism was normal at 33°C, except for the increased rate[5,7].

## Wholemount Brightfield and Fluorescent Imaging

Live embryos and adult zebrafish were anesthetized using 2-phenoxyethanol prior to microscopy. Images were taken on a Spot 2 digital camera (Diagnostic Instruments; Sterling Heights, MI) attached to a Leica MZFL III stereomicroscope, with the Leica GFP3 filter for EGFP fluorescent images. All images were edited with Adobe Photoshop 7.0.

## Immunolabeling and Confocal Imaging

Following various lengths of time of regeneration, fins were reamputated proximal to the original amputation plane and fixed overnight at 4°C in a solution of either 4% paraformaldehyde/5% sucrose/1X PBS (for BrdU immunostaining) or 9:1 ethanolic formaldehyde (100% ethanol:37% formaldehyde, for PCNA and EGFP immunostaining). Following fixation, fins were washed (3X, 20 min) in 5% sucrose/1X PBS. Samples were embedded in a heated (55°C) solution of 5% sucrose/1.5% agar. The tissue was cryoprotected through a series of washes for 10 min each in 30% sucrose, 30% sucrose/TBS, TBS alone. Blocks of fin tissue were embedded in Tissue Freezing Medium (Triangle Biomedical Sciences, Durham, NC) and sectioned at 14 μm. The frozen sections were dried for 2 h at 50°C and then rehydrated in 1X PBS.

EGFP immunolocalization was performed on fins that were fixed in 9:1 ethanolic formaldehyde for either PCNA coimmunolocalization or following RNA wholemount *in situ* hybridization (see next section). Proliferating cell localization was assessed using PCNA immunostaining on sectioned fins as described[17]. Briefly, sections were incubated in blocking solution (1X PBS/2% normal goat serum/1% DMSO/0.2% Triton-X 100) for 2 h at room temperature and then overnight at 4°C in 1:1500 dilution of a rabbit anti-GFP polyclonal antibody (Abcam; Cambridge, MA) and a 1:1000 dilution of mouse monoclonal anti-PCNA antibody (clone PC10, Sigma Chemical; St. Louis, MO) in blocking solution. The sections were washed in 1X PBS/0.05% Tween-20 and then incubated for 1 h at room temperature in 1:500 Alexa Fluor 594 goat antimouse secondary antibody (Molecular Probes; Carlsbad, CA) and 1:500 Alexa Fluor 488 goat antirabbit secondary antibody diluted in 1X PBS/0.05% Tween-20. A nuclear stain, TO-PRO-3 (Molecular Probes), was added to the secondary antibody solution at a 1:750 dilution. Sections were washed in 1X PBS/0.05% Tween-20, 1X PBS and covermounted using ProLong Gold (Molecular Probes). PCNA-positive cells (red nuclei) and EGFP-positive cells (green cells) were analyzed using confocal Z-stack images (depth 10 μm, 1 μm per stack) obtained on a 1024 BioRad confocal microscope.

## RNA *In Situ* Hybridization

Wholemount *in situ* hybridization was performed as previously described on 24 hpf embryos[43] using a digoxigenin-labeled antisense *msxc* probe[44]. Following detection of the *msxc* transcript, fins were postfixed in 9:1 ethanolic formaldehyde and processed for cryosectioning as described above. EGFP

immunolocalization was preformed on cryosections as described above and images were obtained on a 1024 BioRad confocal microscope.

### Bisulfite Mapping of Methylated and Nonmethylated DNA

Genomic DNA was isolated from the Tg(*ef1-α:EGFP*)$^{nt}$ line as previously described[35]. An aliquot of 0.5 μg of genomic DNA per sample was subjected to bisulfite conversion using the EZ DNA Methylation-Gold Kit (Zymo Research; Orange, CA). To convert all the nonmethylated cytosines to uracils, 130 μl of CT conversion reagent was added to each DNA sample and incubated in a thermal cycler at 98°C for 10 min, 64°C for 2.5 h, and then 4°C for 20 h. The methylated cytosines fail to react and do not become uracils. The converted DNA was purified using a Zymo-Spin IC column and desulfonated following the protocol in the kit. A yield of approximately 25 ng/μl (250 ng total) was achieved for each sample after column purification. A ~ 300-bp product, which included EGFP's translational start site, was PCR-amplified using Platinum Taq High Fidelity enzyme (Invitrogen) from 125 ng of converted DNA with the following two sets of converted primers: set 1, forward 5' – TTGAGATGAGGATAAAATATTTGAG – 3' and reverse 5' – TAATACAAATAAACTTCAAAATCAAC – 3'; set 2, forward 5' GGTTGTTGTGTTGTTTTATTATTTTGG – 3' and reverse 5' – ATAACTATTATAATTATACTCCAACTTATACC – 3'. The cycling conditions were as follows: denaturing at 94°C for 2 min, followed by 35 cycles of 94°C for 30 sec, annealing at 52°C for 1 min, extension at 68°C for 8 min followed by a single incubation at 68°C for 10 min at the conclusion of the 35 cycles. PCR fragments were cloned into the TOPO pCR4 vector (Invitrogen) and sent to Sequetech, Inc (Mountain View, CA) for sequencing. The 300-bp pT2KXIG contains either 19 or 17 potential CpG islands (primer sets 1 and 2, respectively), which were scored as nonmethylated if a TG was present and the CpG island (C converted to U) or methylated if the CG was still present at a CpG island. A total of six clones using the first set of primer pair and four clones using the second primer pair were amplified, sequenced, and analyzed.

### Constant Light Treatment

Light treatment was performed as previously described[17,18,29]. Briefly, 6- to 9-month-old *albino* Tg*(ef1-a:EGFP)*$^{nt}$ zebrafish were dark-adapted for 7 days and then transferred to 2 l clear polycarbonate tanks and placed between four 150-W halogen lamps. Two lamps directly flanked the tank (50 mm from the center of the tank) and two additional lamps were placed at 45 degrees above the tank (70 mm from the center of the tank). This arrangement generated a light intensity of 3,500 lux in the tank. The temperature of the room was tightly regulated such that water temperature did not exceed 33°C. Fish were light treated for 36 continuous hours, at which point they were euthanized and their eyes harvested and processes for retinal analysis.

## ACKNOWLEDGMENTS

We would like to thank Koichi Kawakami for the Tol2 plasmid, pT2KXIG, and the Tol2 *transposase* gene, and Debbi Bang and the Freimann Life Science staff for general zebrafish husbandry.
